# Effects of clinical interventions through a comprehensive medication management program: A retrospective study among outpatients in a private hospital

**DOI:** 10.1016/j.rcsop.2024.100440

**Published:** 2024-04-04

**Authors:** Esteban Zavaleta-Monestel, Bruno Serrano-Arias, Sebastián Arguedas-Chacón, Alonso Quirós-Romero, José Pablo Díaz-Madriz, Arturo Villalobos-Madriz, Allan Robles-Calderón, Jorge Bucknor-Masís, José Miguel Chaverri-Fernández

**Affiliations:** a Pharmacy Department, Hospital Clínica Bíblica, 1307 San José, Costa Rica; b Internal Medicine Department, Hospital Clínica Bíblica, 1307 San José, Costa Rica; c Faculty of Pharmacy, Universidad de Costa Rica, 11801 San José, Costa Rica

**Keywords:** Pharmaceutical care program, Comprehensive medication management program, Pharmaceutical interventions, Drug-related problem

## Abstract

**Introduction:**

The intricate nature of certain diseases necessitates complex medication regimens, utilization including high-cost medications, and continual vigilance to avoid potential complications. To address these exigencies, numerous healthcare institutions have instituted multidisciplinary management teams, exemplified in pharmaceutical care through Comprehensive Medication Management (CMM) programs. These programs oversee diverse facets such as patient education, medication adherence promotion, clinical monitoring, dose adjustments, and scrutiny of prescribed drug therapies. Given the emphasized significance, it is relevant to possess evidence to continue endorsing these initiatives from management positions within health centers, and it is for this reason that this study aims to evaluate the clinical and economic benefits provided by a CMM program within a private hospital in Latin America, by analyzing the effects of clinical interventions.

**Methods:**

A retrospective examination was conducted involving documented pharmaceutical interventions in an outpatient setting from January 2019 to September 2022. To assess the interventions' repercussions, a retrospective analysis was undertaken. The collated data included patients' basic characteristics, a comprehensive pharmacist-generated description of interventions, potential associated complications, and avoided medical services. Multiple clinical projections, which were endorsed by internal medicine physicians, were developed to explore potential scenarios in the absence of pharmaceutical care. These projections were associated with conceivable complications, aligned with the most plausible circumstances. Subsequently, utilizing the average cost of healthcare within a private hospital in Latin America, the cumulative savings were quantified. These savings were then attributed to the intrinsic advantages offered by pharmaceutical care.

**Results:**

The study discloses demographic trends among patients within distinct age groups in the CMM program. Rheumatology predominated as the main referral source, and interventions centering on monitoring emerged as the pivotal drug-related concern. This encompassed a collaborative approach, involving interdisciplinary efforts toward patient education and critical parameter monitoring. Of the total 347 pharmaceutical interventions, 66.3% (*N* = 230) specialty office visits, 14.1% (*N* = 49) general practitioner consultations, 12.4% (*N* = 43) hospitalizations, and 7.2% (*N* = 25) ER visits were avoided. The economic analysis underscores cost savings ensuing from pharmaceutical interventions, amounting to a cumulative 603,792.82 USD. Extrapolating these findings to a patient cohort of 400 enrolled in the pharmaceutical care program approximates per-patient savings of 361.47 USD.

**Conclusion:**

This study reveals the significant clinical and economic benefits of CMM programs, led by multidisciplinary pharmaceutical professionals. The findings provide compelling evidence for hospital management to consider promoting such programs, drawing from the patient-centered care model in the United States applicable to Latin America.

## 1 Introduction

In the realm of healthcare, medications play a central role, constituting a critical component in approximately 80% of treatment plans.^1^^,^^2^ The significance of medication-related issues becomes even more pronounced when considering the scale of prescriptions dispensed, projected to approach 5 billion in the United States by 2021.^2^ This staggering figure signifies an escalation of roughly 1 billion prescriptions within a mere decade.^2^ Amid this context, the World Health Organization's data underscores the challenge of therapy adherence, revealing an average adherence rate of 50% for chronic diseases in developed nations, the consequences of suboptimal adherence are far-reaching, encompassing compromised health outcomes and an escalation in healthcare expenditures.^1^^,^^2^

In 2017 prevention of drug-related harm was established as a point of interest, consequently, the objective of preventing it was stated as a safety goal by international authorities.^1^^,^^2^ Therefore, drug-related problems (DRPs) can be defined as events or circumstances involving medication that may interfere with desired health outcomes.^1^^,^^2^ Some authors attribute the capability of reducing DRPs to pharmacists who provide pharmacotherapeutic follow-ups through patient education, pharmacovigilance, and drug information.^1^ To achieve an integral analysis of patients, comprehensive medication management (CMM) programs were designed to conduct a thorough assessment of patients, aiming to enhance the efficiency, safety, and compliance of their medication routines, all while mitigating the risk of adverse effects and drug interactions.^1^^,^^3^

The steps, which originated from the proposal of The American College of Clinical Pharmacy (ACCP) as a standard of care to address medication-related problems and ensure the well-being of each patient, have been integrated into the concept of CMM programs.^1^^,^^2^ These plans are designed to tailor therapy for patients dealing with complex polypharmacy situations.^1^ Typically, the analysis of CMM consists of four sequential stages. It commences with an assessment of potential medication-related concerns that patients might have. Subsequently, there is an evaluation of the prescribed regimen, considering factors such as safety, effectiveness, and alignment with the specific drug indications.^3^ Following this evaluation, a plan is put into action to optimize drug therapy. The final step involves consistent monitoring of the outcomes resulting from these therapeutic strategies.^2^

The development of similar programs has been done in different countries of Latin America. In the case of Costa Rica it has been recently implemented in the public and private sectors, where pharmacotherapeutics follow-ups, and where medication compliance enhancement is one of the main focuses of interest.^4^ Improve medication compliance through pharmaceutical follow-ups refers to education aimed at patients and their support circle, so the prescribed regimen can be followed accordingly, this has an important relation with the evaluations of CMM programs through outcomes in diabetes and hypertension patients, as medication compliance in these follow-ups are involved as well.^5^ Therefore, the purpose of the pharmacist-led CMM program is based on the evidence that favors this initiative in outpatient clinics and showed a significant improvement in positive outcomes in drug therapy.^6^^,^^7^ The development of CMM has been a growing field through which pharmacists can provide patients with pharmacotherapeutic follow-ups and help them achieve better outcomes in the evolution of their disease.^4^

The implementation of the CMM program was initiated by the Hospital's Pharmacy Department by a clinical pharmacist. The primary objective of this program was to offer comprehensive follow-up care to ambulatory outpatient patients with complex medical needs. The patient cohort enrolled in this program predominantly comprises individuals with intricate medical conditions or a notable burden of comorbidities, leading to a requirement for a sophisticated and multifaceted drug treatment regimen.

Given the susceptibility of polypharmacy (people using five or more different drugs at the time) patients to issues like medication non-adherence, adverse drug reactions, and drug interactions, the pharmaceutical consultations provided by this program are strategically designed to enhance medication adherence and mitigate the occurrence of medication-related complications.^8^^,^^9^

Furthermore, this involves patient education, particularly in cases involving high-cost medications that necessitate subcutaneous administration, such as biological therapy. Notably, several pharmaceutical industries have expressed interest in leveraging the expertise of pharmacists to educate patients on the proper utilization of their specialized products.^10^^,^^11^

Given the emphasized significance, it is relevant to possess evidence to continue endorsing these initiatives from management positions within health centers. This study aims to assess the clinical and economic benefits of a CMM program in a private Latin American hospital. Specifically, to analyze the effects of clinical interventions on patient outcomes, potential complications avoided, and associated cost savings.

## 2 Methods

### 2.1 Study design

This study is characterized by a retrospective design, which examined pharmaceutical interventions conducted within a specified time frame. These interventions were part of the CMM program, which aimed to optimize patient outcomes by addressing medication challenges through a holistic approach. The components of this program included thorough medication reviews, interventions, and collaboration among healthcare professionals. The goals were to improve medication effectiveness, minimize adverse effects, and enhance patient adherence. The process encompassed a structured assessment, regular reviews, adjustments, patient education, and continuous monitoring to tailor medication plans and improve overall healthcare outcomes.^1^^,^^2^

### 2.2 Setting

The study was conducted in the outpatient setting of a private hospital. This program involved the active management of clinical records and the implementation of interventions designed to mitigate medication-related issues. The CMM program recruits patients who may potentially benefit from its services or acquires patients through referrals from other healthcare professionals. Patients enrolled in the program undergo monthly or weekly monitoring related to their medication, addressing any concerns, and are closely supervised for potential side effects, therapeutic failure, or decreased adherence.

### 2.3 Inclusion criteria

The study includes patients who are engaged in CMM interventions. To be eligible for participation in the study, patients must meet certain criteria, one of which involves being categorized as polypharmacy patients (individuals taking or using more than 5 distinct medications) within the program. Patients have several pathways to join the program. They can proactively initiate their involvement by approaching the healthcare center after learning about the initiative through various communication channels or advertisements. Moreover, patients can be directly referred to the program by other healthcare professionals operating within the hospital environment. Additionally, the pharmacist in charge of the program actively identifies patients retrospectively with past hospital discharges who fit a profile in which they would benefit from being included in the project. These interventions cover the timeframe from January 2019 to September 2022.

### 2.4 Exclusion criteria

Interventions with incomplete or insufficient data are excluded from the study. Furthermore, interventions considered irrelevant, duplicated, or incomplete, along with those addressing a singular drug-related issue through multiple interventions, are excluded from the analysis.

### 2.5 Sample

The sample comprises patients enrolled in the CMM program. Among this population, the study explores the outcomes of a total of 763 documented pharmaceutical interventions. These interventions have been registered for 400 patients, with certain patients receiving multiple interventions. The patients included in the study were followed up as required by the clinical needs of each case in subsequent appointments.

### 2.6 Data analysis

Initially, a raw database prepared by the pharmacist in charge of the CMM included the following information:

a) Date of the pharmaceutical intervention.
b) Patient's age.
c) Patient's gender.
d) Patient's initials.
e) Referring to medical service.
f) A person intervened by the pharmacy professional.
g) Medication-related needs (Adherence, Effectiveness, Necessity, Safety).
h) Classification of Negative Medication Risk (Very high or low dose, presence of adverse effects, need for additional monitoring, Adherence, Need for additional pharmacological therapy, ineffectiveness of selected therapy, unnecessary medication).
i) Cause of the problem.
j) Description or context in which the pharmaceutical intervention took place.
k) Pharmaceutical intervention performed.

Data cleaning was necessary, primarily due to significant missing information, especially in the description of the intervention. Interventions with missing information in this regard were excluded. Similarly, interventions performed on minors were excluded from this analysis. An important limitation of this information is the lack of patient file numbers or identifying information, making it impossible to obtain their list of ailments, medical history, or medication lists. Therefore, the description of the pharmaceutical intervention became the most crucial parameter for determining which interventions were viable. Some descriptions were highly specific, revealing the patient's ailments, the treatment involved, and the manifestation of the problem. Others were as nonspecific as reinforcing therapeutic adherence measures, but without knowledge of ailments or treatments, such interventions were eliminated. After eliminating all interventions with missing information, a final total of 347 interventions were available for analysis over the period from January 2019 to September 2022.

Subsequently, these data were reorganized according to the Classification of Negative Medication Risk. Segmenting the data in this way facilitated the analysis of each intervention according to the desired outcome. From this, it was possible to illustrate the prevalence frequencies of these events, as shown in Table 2.

For each case individually, an external pharmacist to the CMM conducted a clinical projection of what the outcome for the patient would have been if the pharmaceutical intervention had not been implemented, presenting possible scenarios. Subsequently, this was validated by two internal medicine physicians who ensured that, according to the description in the context of each intervention, the projected outcome was indeed the most plausible.

### 2.7 Costs estimation

The possible complication and medical service avoided was proposed by the clinical pharmacist of the CMM program, but eventually, this was validated by two internal medicine physicians. The result of this validation is a total number of physician office visits, ER visits, and hospitalizations avoided, according to the evaded complications due to pharmaceutical interventions. For each one of these medical services, there's an average cost associated, this was calculated based on the registered costs of these services in a private hospital in Costa Rica in 2021. The hospitalization event was associated with the average days of stay obtained. From Hospital records for estimation. Through this data, it was then possible to calculate the total savings cost by reproducing other authors' calculations.^2^^,^^12^

Using this dataset, it became feasible to compute the overall cost savings (using Formula 1), wherein ACS denotes the average cost of service at the health center, and NC signifies the number of cases.^2^^,^^12^

(1) $$\sum_{i=1}^{n}\left(\mathrm{ACS}*\mathrm{NC}\right)\;$$

## 3 Results

The study findings reveal significant patient demographic patterns within age groups in the CMM program. Rheumatology led medical referrals to the program and monitoring interventions emerged as the most addressed drug-related issue, involving interdisciplinary collaboration for patient education and critical indicator identification. The economic analysis showcases cost savings resulting from pharmaceutical interventions. This data, sourced from internal medicine practitioners, enables tailored cost calculations based on individual patient contexts. The method involves avoiding hospitalization days, revealing substantial cumulative cost savings during the study timeframe.

Table 1 delineates the foundational demographic characteristics of patients incorporated within the CMM program who constituted the study cohort, wherein it is noteworthy that 64.3% (*N* = 223) of patients fell within the age brackets of 40–64 and 65–79.

**Table 1:** Basic demographic characteristics of patients enrolled in the CMM. (*N* = 347).

Patient Characteristics n (%)	
*Gender*	
Male	118 (34.0)
Female	229 (66.0)
*Age (years)*	
18–24	17 (4.90)
24–39	60 (17.3))
40–64	130 (37.5)
65–79	93 (26.8)
80 or older	47 (13.5)
*Intervened*	
Patient	206 (59.5)
Healthcare professional	94 (27.1)
Patient's family or caregiver	47 (13.4)

**Fig. 1:**
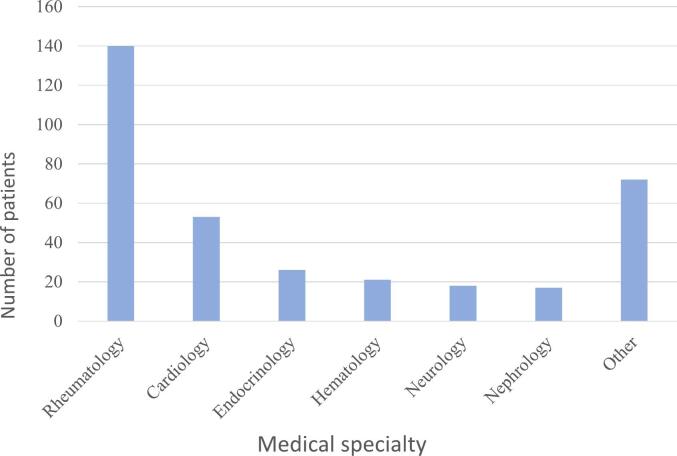
Medical services of referral to the CMM program.

Fig. 1 illustrates the primary medical specialties that refer patients to the CMM program. Rheumatology emerges as the leading source of patient referrals, followed by the “other” category, which includes specialties listed in descending order based on the number of referred patients: Geriatrics, Dermatology, Internal Medicine, Neurosurgery, Pulmonology, Psychiatry, Gastroenterology, Infectious Diseases, General Medicine, Gynecology, Oncology, and Otorhinolaryngology.

In Table 2, the breakdown of a total of 347 documented interventions during the study period is presented based on the classification of drug-related problems. The most frequently reported intervention was the need for monitoring. This category is noteworthy for its interdisciplinary collaboration, wherein the pharmacist, in conjunction with attending physicians, elaborated a plan to educate the patient and identify or pay attention to warning signs, laboratory values, or other pertinent variables associated with the patient's pharmacotherapy.

**Table 2:** Classification of drug-related problems.

Category of Drug Therapy Problems		Number of Drug Therapy Problems n (%)
Adherence	Adherence	77 (22.19)
	Costs	2 (0.58)
Effectiveness	Dosage too low	40 (11.53%)
	Ineffective Drug	20 (5.76%)
	Requires monitoring	97 (27.95%)
Indication/Effectiveness	Unnecessary drug	13 (3.75%)
	Requires additional drug therapy	19 (5.48%)
Safety	Dosage too high	9 (2.59%)
	Requires monitoring	16 (4.61%)
	Adverse drug reaction	54 (15.56%)

Finally, the outcomes of the economic analysis are presented in Table 3. It specifically illustrates the total cost savings resulting from the pharmaceutical interventions in each case examined. To estimate the number of events prevented by the interventions, the data provided by pharmaceutical professionals was validated by both internal medicine physicians, based on the information obtained from the intervention records within the CMM service. This procedure unveiled cumulative cost savings equivalent to 603,788.85 USD over the defined study period.

**Table 3:** Estimated saving costs through the interventions generated in the CMM.

Potential Medical Services avoided	Number of events	Estimated costs per event in USD	Total Savings in USD
Clinic outpatient visit	49	46.82	2293.69
Specialty office visit	230	121.71	27,993.30
ER visit	25	233.84	5846.00
Hospitalization	43	3262.39	567,655.86

## 4 Discussion

The initial demographic analysis, based on the results presented in Table 1, reveals a predominantly female population. These findings depict distributions that align with outcomes observed by other researchers. Furthermore, it is imperative to acknowledge that since the CCM service is not specific to any pathology or target audience, it generates a wide-ranging demographic.^1^^,^^2^ The observation that 64.3% (*N* = 223) of patients fall within the age categories of 40–64 and 65–79 implies a concentration of chronic diseases being addressed by the service.^3^^,^^5^

When contrasting the outcomes depicted in Fig. 1 with the earlier discourse about medical specialties referring patients to the service, it is noteworthy that Rheumatology is the primary source of patient referrals to the service, as opposed to other specialties shown, such as Cardiology, Endocrinology, or Nephrology. This phenomenon can be elucidated by the operational model of the CMM service in this specific hospital, which centers on addressing educational and advisory needs related to high-cost biological medications or targeted therapies, predominantly employed in the management of rheumatic diseases.^10^^,^^11^

The primary population that underwent interventions were the patients themselves. Consequently, measures were implemented that encompassed strategies to enhance adherence, educational plans for initiating new treatments, and training on disease decompensation monitoring, enabling patients to engage in self-monitoring for subsequent evaluation during follow-up periods. Interventions directed at healthcare personnel encompassed a range of actions, with the most common being the development of a multidisciplinary plan that ensures the prescribing physician is also cognizant of specific monitoring details related to treatment safety, efficacy, and pharmacological necessity.^4^^,^^13^

The data presented in Table 2 underscore the significance of pharmaceutical interventions within this population. In addition to monitoring events related to patients' pharmacotherapy, the table reveals that interventions concerning adherence strategies constitute a substantial portion of the total interventions. This is pertinent due to the global evidence of low treatment adherence, making pharmacist interventions in this regard a valuable contribution that has demonstrated a positive impact on patient clinical outcomes.^1^^,^^2^ This confirms that clinical pharmacists, aside from devising educational strategies, possess the capability to effect clinical change through interventions about dosing, modification, and optimization of pharmacological therapies, following a comprehensive model that can be applicable beyond North America.^3^^,^^14^

In connection with the detected or prevented adverse effects, CMM activities encompass the assessment of drug regimen safety, which includes evaluating the risk of adverse drug reactions and implementing measures to prevent them. Some of these adverse events were identified through tasks such as reviewing laboratory reports and conducting clinical evaluations performed by the responsible pharmacist within the program. The likelihood of developing adverse drug reactions is heightened in patients undergoing polypharmacy due to potential drug interactions and heightened sensitivities.^3^

Consequently, various healthcare institutions have recognized the necessity of incorporating clinical pharmacists to oversee the progression of polypharmacy patients. This recognition has prompted diverse clinical centers to express interest in adopting CMM programs.^1^ For instance, in the context of heart failure patients, the introduction of such practices has yielded positive outcomes, marked by a reduction in hospitalization rates. This attests to the substantial advantages of enhancing the network of care for polypharmacy patients.^6^, ^15^, ^16^, ^17^

The outcomes presented in Table 3 were initially formulated by clinical pharmacists and subsequently reviewed or adjusted by two internal medicine physicians. Their task was to exclusively consider the most plausible scenario by the context described in each pharmaceutical intervention. The cost estimation was formulated to approximate the economic valuation of the CMM program. This valuation was drawn from a study conducted in Texas that investigated a similar subject.^12^ The mentioned study revealed that their CMM program achieved cost savings amounting to 1,185,610.00 USD, encompassing a population of 3280 patients over nine months. Consequently, the calculated savings per patient were approximately 361.47 USD.^12^

Applying the same methodology to the available clinical data extracted from the CMM program's patient files, it was determined that the average savings per patient amounted to 1509.47 USD. While the discrepancy between these two values might appear substantial, it's important to consider that both programs operated differently and gathered data over distinct time frames.^12^ The Texas CMM program admitted chronic patients from a clinic, while the program in this study exclusively accepted patients referred from other medical services, where medication-related issues were already prevalent. As a result, nearly every patient received at least one pharmaceutical intervention.^12^

It is crucial to note that directly comparing these two countries in economic terms without some form of adjustment would not yield entirely meaningful results, given the substantial variations in healthcare costs across these regions and other disparities within their healthcare systems, as highlighted by organizations such as The Organization for Economic Cooperation and Development (OECD).^18^^,^^19^ As an illustration, it is worth noting that the Gross Domestic Product (GDP) per capita for the United States in the year 2021 stands at 70,248.6 USD, whereas the corresponding GDP per capita for Costa Rica in the same year is 12,472.4 USD.^18^

The healthcare system in Costa Rica has evolved, primarily driven by social security provisions, thereby facilitating open access to healthcare services for the entire populace without discrimination.^19^ However, despite this theoretically equitable framework, public healthcare institutions find themselves strained due to the substantial volume of individuals seeking care, leading to the gradual emergence of protracted waiting lists. Consequently, numerous individuals have opted to enroll in private insurance plans or avail services from private healthcare facilities, assuming the financial responsibility out-of-pocket, with a noticeable decline in associated costs over the years. This transition is evident from the fact that by 2017, approximately 32% of the Costa Rican population had shown a preference for the private healthcare system.^19^ Considering this context, the implementation of strategic initiatives such as CMM within private hospitals in Costa Rica could potentially yield advantages for the pharmacy profession's continuous expansion. Moreover, these endeavors can equip patients with diverse tools to attain enhanced therapeutic outcomes, exceeding what is conventionally provided by standard healthcare services.^19^

## 5 Strengths and limitations

The main strength of this study is its demonstration of the reproducibility of a CMM methodology within the context of low and middle-income countries. However, there are several limitations to this study. It is essential to acknowledge that this study was conducted at a single center, which might restrict the generalizability of the findings. Additionally, another significant limitation is the sample size. Given that this is a pioneering and retrospective study, the inclusion criteria constrained the enrollment of a relatively small number of subjects. Consequently, the collected demographic characteristics were restricted due to the methodology mentioned. Table 1 displayed restricted information, leaving room for various other potential confounding factors that might have contributed to the occurrence of drug-related issues. Moreover, explicit particulars were not furnished regarding the resolution methods for each drug therapy problem, including associated cost considerations. Lastly, biases exist in the attribution of significance or outcomes about pharmaceutical interventions by these same professionals. Furthermore, biases are also present in the professional judgment exercised by the collaborating physicians within the study.

## 6 Conclusions

In conclusion, our study provides compelling evidence supporting the significant clinical and economic benefits of a pharmacist-led CMM program in the context of a private hospital in Latin America. Furthermore, it underscores the importance of interdisciplinary work and a collaborative approach to maximize the impact of CMMs, resulting in patient benefits, avoidance of medical services, and complications.

## Ethical approval

Approval to conduct this study following ethical standards was secured from the Scientific Ethical Committee of the University of Costa Rica (approval date: 13, 2022), under the reference number CEC284–2022. It is important to note that written consent was not required for this study.

## Declaration of artificial intelligence (AI) in the writing process

During the preparation of this work, the authors used ChatGPT August 3 Version to translate and rephrase text in academic language. After using this tool/service, the author(s) reviewed and edited the content as needed and take(s) full responsibility for the content of the publication.

## CRediT authorship contribution statement

**Esteban Zavaleta-Monestel:** Conceptualization, Investigation, Methodology, Project administration, Supervision, Validation, Visualization. **Bruno Serrano-Arias:** Conceptualization, Data curation, Methodology, Writing – original draft. **Sebastián Arguedas-Chacón:** Conceptualization, Methodology, Project administration, Supervision, Writing – original draft, Writing – review & editing. **Alonso Quirós-Romero:** Data curation, Investigation, Writing – original draft. **José Pablo Díaz-Madriz:** Conceptualization, Project administration. **Arturo Villalobos-Madriz:** Conceptualization, Project administration. **Allan Robles-Calderón:** Data curation, Formal analysis, Supervision. **Jorge Bucknor-Masís:** Formal analysis, Methodology, Supervision. **José Miguel Chaverri-Fernández:** Conceptualization, Investigation, Supervision.

## Declaration of competing interest

The authors declare that they have no known competing financial interests or personal relationships that could have appeared to influence the work reported in this paper.
